# Vestibular Schwannoma Management: Patient Perspectives and Health Literacy

**DOI:** 10.1097/ONO.0000000000000054

**Published:** 2024-04-26

**Authors:** Julia A. Casazza, Kristen L. Yancey, Jacob B. Hunter

**Affiliations:** 1 Department of Otolaryngology, Medical School, University of Texas Southwestern Medical Center, Dallas, TX; 2 Department of Otolaryngology-Head and Neck Surgery, Weill Cornell Medicine, New York, NY; 3 Department of Otolaryngology-Head and Neck Surgery, Thomas Jefferson University, Philadelphia, PA.

**Keywords:** Health literacy, Loss to follow-up, Patient perspectives, Vestibular schwannoma

## Abstract

**Objective:**

To assess medical decision-making, reasons for attrition, and health literacy among vestibular schwannoma (VS) patients last seen in clinic more than 2 years ago.

**Study Design:**

Survey.

**Setting:**

Tertiary skull base center.

**Patients:**

Adults with sporadic VS last evaluated more than 2 years ago.

**Methods:**

Survey including Brief Health Literacy Screen (BHLS), a validated 3-question measure of health literacy, and open-ended questions on care; retrospective chart review.

**Main Outcome Measures:**

BHLS score, patient reasons for attrition.

**Results:**

Of 1011 patients contacted, 205 (20.3%) patients responded and met all criteria for analysis. At initial evaluation, mean patient age was 50.6 ± 12.8 years, roughly half (51.4%) were female, and the majority (80.2%) identified as White. At the time of the survey, 48 (23%) continued to observe their tumor. The remaining 160 (76.9%) patients previously underwent treatment (surgery and/or radiation). Of those treated, 34 (21.3%) underwent intervention elsewhere. Symptoms since the last patient visit did not differ between observed and treated patients. About 94.7% of the cohort displayed high health literacy (BHLS > 9), though rates of inadequate health literacy were higher among observed patients (12.5% versus 3.1% in those treated). The most common reasons for opting not to follow-up included counseling issues, personal decisions, and social/life stressors (including the COVID-19 pandemic).

**Conclusions:**

Despite being a highly health-literate cohort, patients cited multiple reasons for attrition. Regardless of whether they were observed or treated, patients described follow-up visits as burdensome and perceived them to be of minimal benefit.

Vestibular schwannoma (VS) management can include surgical resection, radiation to arrest growth, or observation with serial imaging. This last option has increased in popularity over the years. In the late 1990s, 7% of patients elected observation as initial management, relative to 33% diagnosed in the 2010s (1). While treatment strategies continue to evolve, most studies of VS have follow-up ranging from 22 to 47 months (2–4), limiting understanding of the disease’s natural history over time.

Regardless of the treatment chosen, lifelong surveillance is traditionally recommended. Surgical patients can experience recurrence (or growth of residual tumor) and tumor progression or malignant transformation can occur following radiation (5). Similarly, stable tumors can enlarge after a period of quiescence (6). Despite these concerns, continued surveillance is challenging. Raymond et al found that only 3 of 122 patients observing their tumors completed appropriate surveillance imaging over the course of 5 years, with a mean follow-up of just 5 months. Neither demographic nor medical factors correlated with a lack of continued imaging (7). Social constraints, health literacy, and personal preference likely influence treatment decisions, but research investigating these contributors is scarce. Additionally, the lack of long-term data on observational management limits a complete understanding for patients and clinicians. While estimates suggest that 40% of tumors demonstrate further growth based on linear dimensions (2), or 18–73% demonstrate growth when considering volumes (8–10), eventual intervention in many of these patients skews long-term natural history statistics.

Understanding why VS patients do not consistently follow in clinic would enable a better understanding of the disease and could improve patient care. Without knowledge of how patients fare long-term following intervention or with observation, recommending a particular management strategy is challenging. To explore the reasons why patients do not adhere to recommended surveillance plans, we surveyed VS patients who had not been evaluated in at least 2 years.

## METHODS

### Patient Selection

This work was performed with approval from the institutional review board (STU 2022-0209). The study consisted of a survey and chart review of adult VS patients (aged 18 years and older), previously evaluated at our institution in 2003 or later, who had not been seen for at least 2 years at the time of chart review in April 2022. Patients were identified from a prospectively maintained Research Electronic Data Capture (REDCap) database using International Classification of Diseases codes for “VS” or “benign neoplasm of cranial nerves” (11,12). Patients with neurofibromatosis type II were excluded. Demographic data reviewed included race, date of birth, age at diagnosis, marital status, and employment. Participant Zone Improvement Plan Code (ZIP) codes were used to calculate a street-level driving distance to our clinic building using FreeMapTools (13). Median household income for ZIP codes was retrieved from Census Bureau data (14). Tumor size (greatest linear axial diameter on MRI) and audiometric data (ipsilateral pure tone averages [PTA; defined as the average of 0.5, 1, 2, and 3 kHz] and ipsilateral word recognition scores [WRS]) were also reviewed.

### Institutional Practice Patterns

Recommended MRI surveillance intervals differ by the elected management. Patients who choose observation are typically recommended to repeat imaging 6 months following the initial diagnostic MRI, followed by annual scans for the first 5 years. If no growth is observed at 5 years, the imaging interval increases to every 2 years. At the 10th year of no growth, scans are then obtained every 5 years. In patients who have undergone surgery, MRIs are ordered between 3 and 12 months postoperatively, depending on the extent of resection and surgeon preference. Radiated patients are recommended for annual MRIs, eventually spacing studies out about 3 years posttreatment.

### Brief Health Literacy Screen (BHLS) and Symptom Survey

All eligible patients were emailed an invitation to participate in the survey, with 2 follow-up reminders. Two attempts were made by phone if patients did not respond to emails or did not have an email address listed in the electronic medical record. Participant health literacy was assessed using the Brief Health Literacy Screen (BHLS), a validated, 3-question instrument answered on a 5-point Likert scale. Higher scores indicate greater comfort in interacting with medical information and making healthcare decisions (Supplemental Appendix A, http://links.lww.com/ONO/A26) (15). A total score less than or equal to 9 indicates inadequate health literacy (16). In addition to the BHLS, participants were queried about the development or progression of symptoms since their last evaluation, with one 3-part question (“Have you experienced any of the following since you were last seen: decline in hearing, decline in balance/dizziness, or new facial weakness/numbness/spasms?”). Patients were also asked to provide reasons for not having been seen in ≥2 years via a free-text response.

#### Coding of Survey Responses

Free-form survey responses to the question “what are your reasons for not returning?” were deidentified, reviewed, and coded by K.L.Y. and J.B.H. Differences in classification were resolved through discussion. Patients who informed us that they had seen clinicians outside our institution in the past 2 years or patients who were following on a longer interval as advised by their clinician were considered to be adherent to surveillance care and thus excluded from the analysis.

### Statistical Analysis

Continuous variables were described with means and SDs, while categorical variables were listed as percentages and ranges. Descriptive statistics were calculated, with 2-tailed student *t* tests used to assess continuous variables and chi-squared/Fisher tests used to assess categorical variables. All statistics were computed with R version 4.1.0 (R Foundation for Statistical Computing, Vienna, Austria) with *P* values <0.05 considered statistically significant.

## RESULTS

**FIG. 1.**
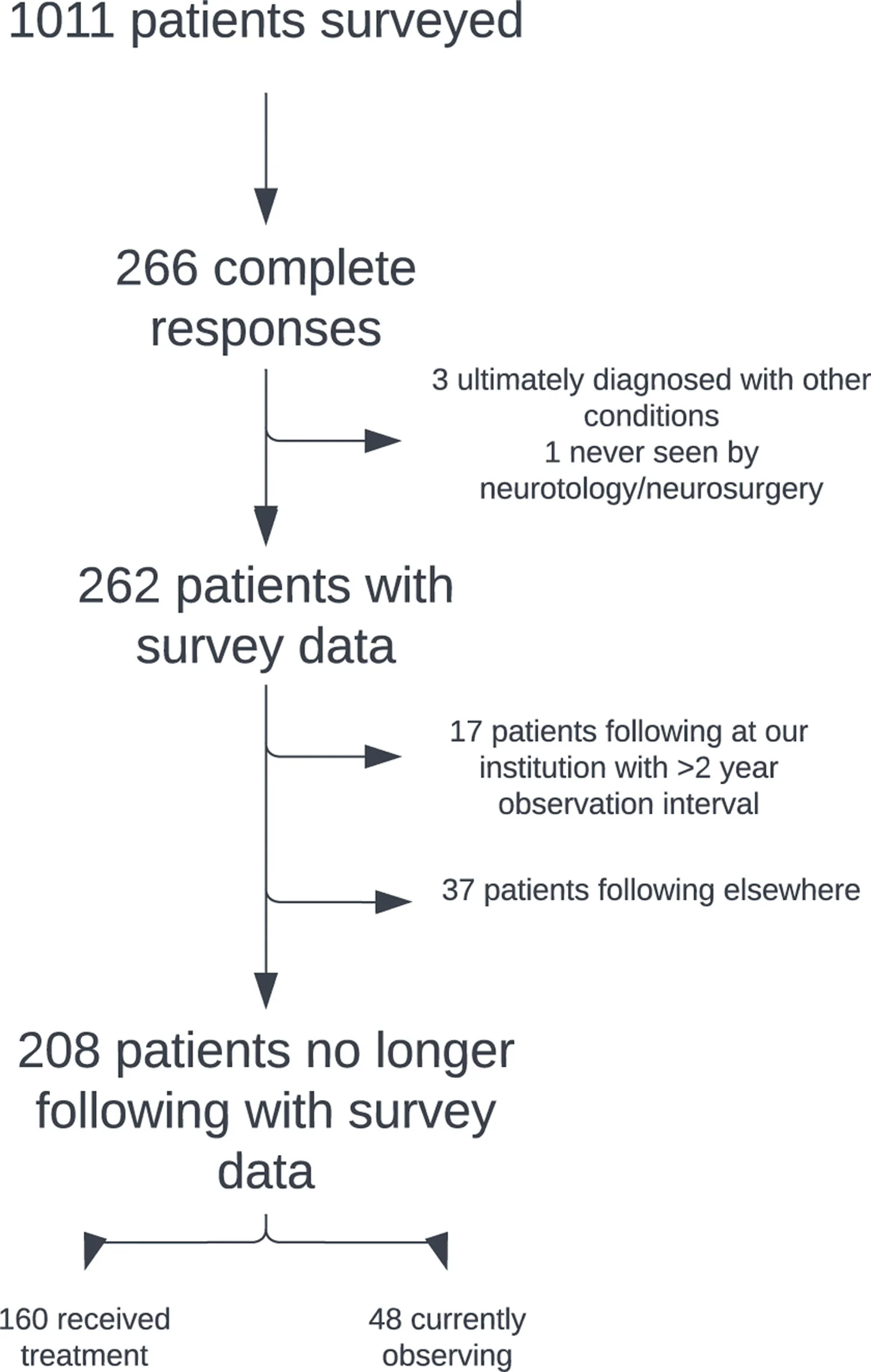
Patients included in study. Of 1011 patients previously evaluated but not seen in clinic over the past 2 years, 208 responded and met all inclusion criteria for our study.

The database review identified 1011 patients last seen at our institution more than 24 months ago, 266 (26.3%) of whom responded to the survey. We excluded 3 patients diagnosed with other conditions, 1 patient who had never been evaluated, 17 (6.4%) patients who were following on a greater-than-2-year interval as specified by their doctor, and 37 (13.9%) patients who transferred care to outside clinicians that they routinely follow with (Fig. 1). Our analysis thus included 208 patients, 160 (76.9%) of whom underwent intervention (surgery and/or radiation), and 48 (23.1%) who were observing their tumors. Telehealth surveillance visits—occasionally arranged at clinician discretion—counted toward patient follow-up.

Of 160 previously treated patients, 122 (76.2%) had surgery, 37 (23.1%) had radiation, and 1 (0.7%) underwent an unspecified treatment. And 34 (21.3%) underwent intervention elsewhere. Treated and observed groups were demographically similar, though treated patients were older at diagnosis (Table 1; mean age ± SD: 54.5 ± 11.8 years versus 49.4 ± 12.9 years; *P* = 0.012). Driving distance to clinic, median income of home address ZIP code, average length of follow-up in our clinic, and time since last appointment did not differ between groups. Median income of home address ZIP code did not differ between groups (Table 1).

**TABLE 1.** Characteristics of treated and observation vestibular schwannoma patients

	Treated (n = 160)	Observation (n = 48)	*P* value
Age at diagnosis, mean (SD), y	49.4 (12.9)	54.5 (11.8)	0.012 95% CI: (−9.1 to −1.1)
Gender, n (%)			
Male	78 (48.8%)	23 (47.9%)	0.919
Female	82 (51.2%)	25 (52.1%)	
Racial/ethnic background, n (%)			
White	128 (80.0%)	39 (81.3%)	>0.99
Black	2 (1.3%)	1 (2.1%)	
Hispanic	6 (3.8%)	1 (2.1%)	
Asian	4 (2.5%)	0 (0.0%)	
Other	2 (1.3%)	2 (4.2%)	
Unknown	18 (11.3%)	5 (10.4%)	
Health insurance, n (%)			
None	7 (4.4%)	2 (4.2%)	0.596
Private PPO	75 (46.9%)	22 (45.8%)	
Private HMO	22 (13.8%)	7 (14.6%)	
Private other	9 (5.6%)	1 (2.1%)	
Medicare	38 (23.8%)	15 (31.3%)	
VA	6 (3.8%)	0 (0.0%)	
Managed care	1 (0.6%)	0 (0.0%)	
Other	0 (0.0%)	1 (2.1%)	
Unknown	2 (1.3%)	0 (0.0%)	
Marital status, n (%)			
Single	19 (13.6%)	5 (10.4%)	0.308
Married	121 (86.4%)	35 (72.9%)	
Widowed	5 (3.6%)	4 (8.3%)	
Divorced	13 (9.3%)	2 (4.2%)	
Lives with partner	1 (0.6%)	1 (2.1%)	
Unknown	1 (0.6%)	1 (2.1%)	
Median household income of home address ZIP code, n (%)			
$20,000 and below	2 (1.3%)	0 (0%)	0.358
$20,000–44,999	14 (8.8%)	8 (16.7%)	
$45,000–99,999	111 (69.4%)	27 (56.3%)	
$100,000–149,999	26 (16.3%)	9 (18.8%)	
$150,000–199,999	1 (0.6%)	0 (0.0%)	
$200,000 and above	5 (3.1%)	3 (6.3%)	
Employment status, n (%)			
Unemployed	4 (2.5%)	1 (2.1%)	0.878
Homemaker	7 (4.4%)	1 (2.1%)	
Student	5 (3.1%)	0 (0.0%)	
Retired	22 (14.4%)	5 (10.4%)	
Working	112 (70.0%)	38 (79.2%)	
Unknown	10 (6.3%)	3 (6.3%)	
Mean driving distance from home ZIP to our clinic (SD), miles	120.7 (232.9)	78.6 (114.6)	0.091 95% CI: (−91.1 to 6.8)
Average length of follow-up in our clinic (SD), y	3.5 (3.8)	3.9 (4.0)	0.522
Average time since last clinic appointment (SD), y	7.5 (4.0)	7.1 (3.6)	0.431

CI indicates confidence interval; HMO, Healthcare Management Organization; PPO, Preferred Provider Organization; VA, Department of Veteran’s Affairs; ZIP, Zone Improvement Plan Code.

Overall, progression of hearing loss was the most commonly reported symptom (reported by 49.8% of all patients), followed by a decline in balance (36.1%) and facial weakness (11.6%). Worsening balance and facial weakness since the last clinic visit did not differ between treated and observed patients. Treated patients had significantly worse hearing when compared with observation patients by PTA (ipsilateral mean PTA ± SD = 76.1 ± 40.6 dB versus 42.6 ± 24.2 dB, *P* < 0.001, 95% confidence interval: −45.8 to −21.1). Ipsilateral WRS did not differ between the 2 groups, likely related to the inability to assess WRS in patients with profound posttreatment hearing loss (Supplemental Table S1, http://links.lww.com/ONO/A24). Mean tumor size at the last MRI did not differ between treated patients and observation groups (Supplemental Table S1, http://links.lww.com/ONO/A24).

### Health Literacy

Of 206 respondents, only 11 (5.3%) BHLS scores suggested inadequate health literacy (Table 2; BHLS score ≤9). On average, respondents indicated high levels of independence in meeting their health-related needs. Patients tended to report feeling very confident reading hospital materials, understanding their medical condition, and comprehending written information (Supplemental Table S2, http://links.lww.com/ONO/A25). Between treated (n = 160) and observed (n = 48) survey respondents, BHLS scores indicated inadequate health literacy more often among observed patients (n = 6 [12.5%] versus n = 5 [3.1%], *P* = 0.02; Table 2).

**TABLE 2.** *Patient responses to the Brief Health Literacy Screen (BHLS) indicated inadequate health literacy was more frequently reported among patients that underwent treatment for their vestibular schwannomas (P = 0.02*)

	Treated (n = 160)	Observation (n = 46)	*P* value
Inadequate health literacy (BHLS ≤9)	5 (3.1%)	6 (12.5%)	0.02
Adequate health literacy (BHLS >9)	155 (96.9%)	40 (87.5%)	

Two patients in the observation group failed to complete all 3 BHLS questions.

BHLS indicates Brief Health Literacy Screen.

### Reasons for Lack of Follow-up

Of 208 respondents, 193 (92.3%) patients responded regarding their decision to not seek VS care in the past 2 years. Patients cited 1–3 reasons motivating their decision. Six themes emerged regarding nonadherence (Table 3). In order of decreasing frequency, reasons included: counseling issues (n = 63, 32.6%), lack of perceived benefit (n = 59, 30.6%), social/life stressors (including COVID-19) (n = 30, 15.5%), logistics (n = 27, 14.0%), finances (n = 16, 8.3%), and dissatisfaction with care (n = 10, 5.2%) (Table 4). Reasons for not following up did not differ based on whether patients were being observed or had undergone intervention at the last follow-up (Table 4). However, treated patients were less likely to mention a fear of COVID-19 illness in their response (Table 4; n = 14 [9.6%] versus n = 11 [23.4%], *P* = 0.028).

**TABLE 3.** Six themes identified among patient responses

	Representative responses
Social/life stressor (ie, COVID pandemic, personal/family illness)	“I have been dealing with other health issues which required more attention.”
Logistics (travel to clinic, moving, scheduling difficulty)	“Tired of the long drive.”
Finances (loss of insurance, high deductible)	“I’ve retired and I don’t have health insurance at this time.”
Counseling (misinterpretation or poor communication of follow-up recommendations)	“I didn’t think I needed any follow up care.”
Personal decisions (no perceived benefit in following up)	“Meh.. don’t have a good reason... Resigned myself to it is what it is. Had the surgery.”
Dissatisfaction with care (dislike of MRIs, pursuit of care elsewhere)	“I figured they’d want me to do an MRI, and I hate those things”

COVID indicates coronavirus disease due to SARS-CoV-2.

**TABLE 4.** Patient-reported reasons for lack of follow-up

	Patients reporting reason (n = 193)	Treated patients reporting reason (n = 146)	Observation patients reporting reason (n = 47)
Social/life stressor (eg, COVID pandemic, personal/family illness)	30 (15.5%) Of whom 25 (13.0%) explicitly mentioned COVID-19 in their response	25 (17.1%) Of whom 14 (9.6%) explicitly mentioned COVID-19 in their response	5 (10.6%) Of whom 11 (23.4%) explicitly mentioned COVID-19 in their response
Logistics (travel to clinic, moving, scheduling difficulty)	27 (14.0%)	21 (14.4%)	6 (12.8%)
Finances (loss of insurance, high deductible)	16 (8.3%)	12 (8.2%)	4 (8.5%)
Counseling (unaware of follow-up recommendations)	63 (32.6%)	49 (33.6%)	14 (29.8%)
Personal decisions (no perceived benefit in following up)	59 (30.6%)	44 (30.1%)	15 (31.9%)
Dissatisfaction with care (dislike of MRIs, pursuit of care elsewhere)	10 (5.2%)	9 (6.2%)	1 (2.1%)

Patients reported up to 3 reasons for not following up.

COVID indicates coronavirus disease due to SARS-CoV-2.

## DISCUSSION

Knowledge on patient decision-making in VS is scarce. In a 2019 study, Raymond and colleagues surveyed 26 patients initially observing their VS who were then lost to follow-up (7). Among this group, 11 opted to pursue care elsewhere, 9 misunderstood counseling, 5 intentionally neglected visits, and 1 had difficulty with transportation (7). Similar to Mallory et al, we found that a substantial portion of observed VS patients do not feel they need lifelong follow-up, a sentiment shared by previously treated patients.

Among patients who had not been evaluated in at least 2 years, the most common reason for not returning to clinic was issues with counseling (n = 63, 32.6%). Patients frequently misunderstood the recommended follow-up interval or were unaware they required surveillance. Development of a protocol to identify and prompt follow-up in patients who have not been seen at the intended interval may also improve surveillance. The second most common reason was a lack of perceived benefit (n = 59, 30.6%). Many expressed that, having lived with their condition for multiple years, further care felt like a nuisance, particularly in light of limited therapies for existing hearing loss and imbalance. This may suggest that current counseling is inadequate in conveying the rationale for and value of long-term surveillance. Alternatively, perhaps less frequent follow-up is appropriate for asymptomatic patients. A minority (24; 11.5%) of patients reported worsening facial weakness, an unexpected finding in this patient population. Over half (n = 14) of these patients had previously undergone surgical resection. As these patients had not been evaluated in over 2 years, it is unknown to what degree facial nerve function had declined or whether increasing dissatisfaction over postoperative outcomes influenced responses.

More specific reasons for attrition varied, ranging from the inconvenience of driving to the personal dislike of MRI scans. Much like the 4 in 10 American adults who deferred healthcare due to fear of COVID-19, 25 (13.0%) of our patients cited COVID-19 as a reason for not following up (17). Observed patients mentioned fear of COVID-19 at more than twice the rate of previously treated patients (23.4% versus 9.6%), perhaps reflecting their older age.

Our results also indicate a discrepancy between health literacy and strict adherence to treatment guidelines. Among 208 survey respondents, health literacy (as measured by the BHLS) was high (Table 2; 94.7), particularly when compared with a recent meta-analysis that estimated up to one-third of surgical patients are health illiterate (18). This finding suggests that VS patients treated in a tertiary academic setting are highly health literate and eager to learn about their condition. Though nearly one-fourth of patients may have misinterpreted clinician recommendations for surveillance (Table 4), they tended to indicate confidence with the conclusions they had drawn regarding their intended follow-up. Many reported surprise that they were not compliant with the intended schedule of visits.

While relatively few survey participant scores indicated inadequate health literacy (n = 11), rates of inadequate health literacy were 4× higher among patients electing observation relative to those previously treated (Table 2; 12.5% versus 3.1%, *P*=0.02). Additionally, observed patients represented the minority (22%, n = 48) of survey respondents who no longer followed in clinic. Perhaps observation patients with limited health literacy are at higher risk of attrition. After all, tumor-associated symptoms tend to stabilize over time, and patients may be unaware of the potential for late-appearing complications from progressive growth (eg, hydrocephalus from mass effect of tumor compression of the brainstem).

Alternatively, bias against intervention might cause patients with lower health literacy to elect observation. Clinician counseling might not adequately address patient concerns regarding intervention and its indications. One study assessed the relationship between time spent on hand surgery evaluations and health literacy, finding those with limited health literacy had visits that were 17% shorter than those with adequate health literacy (19). If a similar phenomenon exists in our clinic, patients with limited health literacy might benefit from increased face time with their surgeon to better convey expected—though at times variable—growth patterns and the underlying rationale for continued surveillance long-term regardless of management strategy elected.

Comparing VS with pituitary microadenoma (PM) contextualizes high attrition rates. Like VS, PM is a benign, painless, gradually progressive condition with potential to compromise cranial nerve function. The Endocrine Society recommends patients diagnosed with PM undergo MRI every 1–2 years for 3 years following initial diagnosis; from this point, surveillance should continue “gradually less frequently thereafter” (20). Of 414 PM patients seen at a tertiary care center between 2003 and 2021, only 177 (43%) followed up after their initial appointment. Of those 177 patients, the median follow-up was 4.91 years (95% confidence interval: 3.88–5.32 years) (21). Much like VS patients, PM patients do not engage in long-term follow-up, perhaps due to the slightly obscure, slowly progressive, and often asymptomatic nature of their disease.

Our results must be interpreted in light of study limitations. Many did not respond to the survey; thus, their perspectives are not included. Response bias likely affects our results, as patients might not tell researchers affiliated with their former clinic exactly why they discontinued care. Self-selection bias could also influence results if the individuals engaging in research have higher health literacy than the average clinic patient. Our patient population includes those with commercial insurance plans and Medicare, underrepresents uninsured patients, and completely excludes those with Medicaid (Table 1). Patients with commercial insurance or Medicare are more likely to have the education and time needed to learn about and manage health conditions. Future research should assess whether subjective symptoms correlate with functional status, include a more diverse patient cohort, and assess how face time with clinicians influences decision-making.

## CONCLUSIONS

A variety of reasons explain the loss to follow-up in a highly health-literate cohort of patients with sporadic VS. However, we found that both observed and treated patients consistently described follow-up visits as inconvenient and of negligible utility in the absence of symptoms. Patient counseling may benefit from a more detailed discussion of the rationale for lifelong surveillance in VS management.

## FUNDING SOURCES

Internal departmental funding was utilized without commercial sponsorship or support.

## CONFLICT OF INTEREST STATEMENT

None declared.

## DATA AVAILABILITY STATEMENT

The datasets generated during and/or analyzed during the current study are not publicly available, but are available from the corresponding author on reasonable request.

## Supplementary Material

## References

[1] Chan SA Marinelli JP Hahs-Vaughn DL Nye C Link MJ Carlson ML. Evolution in management trends of sporadic vestibular schwannoma in the United States over the last half-century. Otol Neurotol. 2021;42:300–305. 10.1097/MAO.0000000000002891 33229882

[2] Hunter JB Francis DO O’Connell BP. Single institutional experience with observing 564 vestibular schwannomas: factors associated with tumor growth. Otol Neurotol. 2016;37:1630–1636. 10.1097/MAO.0000000000001219 PMC5102804 27668793

[3] Kim JS Cho YS. Growth of vestibular schwannoma: long-term follow-up study using survival analysis. Acta Neurochir (Wien). 2021;163:2237–2245. 10.1007/s00701-021-04870-8 34003365

[4] Godefroy WP Kaptein AA Vogel JJ Van Der Mey AGL. Conservative treatment of vestibular schwannoma: a follow-up study on clinical and quality-of-life outcome. Otol Neurotol. 2009;30:968–974. 10.1097/MAO.0b013e3181b4e3c9 19730147

[5] Balasubramaniam A Shannon P Hodaie M Laperriere N Michaels H Guha A. Glioblastoma multiforme after stereotactic radiotherapy for acoustic neuroma: case report and review of the literature. Neuro Oncol. 2007;9:447–453. 10.1215/15228517-2007-027 PMC1994102 17704364

[6] Nakatomi H Jacob JT Carlson ML. Long-term risk of recurrence and regrowth after gross-total and subtotal resection of sporadic vestibular schwannoma. J Neurosurg. 2017;133:1052–1058. 10.3171/2016.11.JNS16498 28524795

[7] Raymond M Ghanouni A Brooks K Clark SM Mattox DE. Adherence to long-term follow-up in patients with sporadic vestibular schwannomas managed with serial observation. OTO Open. 2021;5:2473974X211036653. 10.1177/2473974X211036653 PMC8358519 34396030

[8] Stipkovits EM Graamans K Vasbinder GBC Dijk JEV Beek FJA. Assessment of vestibular schwannoma growth: application of a new measuring protocol to the results of a longitudinal study. Ann Otol Rhinol Laryngol. 2001;110:326–330. 10.1177/000348940111000406 11307907

[9] Yamamoto M Hagiwara S Ide M Jimbo M Arai Y Ono Y. Conservative management of acoustic neurinomas: prospective study of long-term changes in tumor volume and auditory function. Minim Invasive Neurosurg. 1998;41:86–92. 10.1055/s-2008-1052023 9651917

[10] Nikolopoulos TP Fortnum H O’Donoghue G Baguley D. Acoustic neuroma growth: a systematic review of the evidence. Otol Neurotol. 2010;31:478–485. 10.1097/MAO.0b013e3181d279a3 20147867

[11] Harris PA Taylor R Thielke R Payne J Gonzalez N Conde JG. Research Electronic Data Capture (REDCap)—a metadata-driven methodology and workflow process for providing translational research informatics support. J Biomed Inform. 2009;42:377–381. 10.1016/j.jbi.2008.08.010 PMC2700030 18929686

[12] Harris PA Taylor R Minor BL; REDCap Consortium. The REDCap consortium: building an international community of software platform partners. J Biomed Inform. 2019;95:103208. 10.1016/j.jbi.2019.103208 PMC7254481 31078660

[13] How Far Is It Between. Available at: https://www.freemaptools.com/how-far-is-it-between.htm. Accessed April 22, 2023.

[14] US Income Statistics - Current Census Data for Zip Codes. Available at: https://www.incomebyzipcode.com/. Accessed May 21, 2023.

[15] Chew LD Bradley KA Boyko EJ. Brief questions to identify patients with inadequate health literacy. Fam Med. 2004;36:588–594. 15343421

[16] Megwalu UC Lee JY. Health literacy assessment in an otolaryngology clinic population. Otolaryngol Head Neck Surg. 2016;155:969–973. 10.1177/0194599816664331 27554512

[17] Czeisler ME Marynak K Clarke KEN. Delay or avoidance of medical care because of COVID-19–related concerns — United States, June 2020. MMWR Morb Mortal Wkly Rep. 2022;69:1250–1257. 10.15585/mmwr.mm6936a4 PMC7499838 32915166

[18] Chang ME Baker SJ Dos Santos Marques IC. Health literacy in surgery. Health Lit Res Pract. 2020;4:e46–e65. 10.3928/24748307-20191121-01 PMC7015264 32053207

[19] Menendez ME Parrish RC Ring D. Health literacy and time spent with a hand surgeon. J Hand Surg Am. 2016;41:e59–e69. 10.1016/j.jhsa.2015.12.031 26880496

[20] Freda PU Beckers AM Katznelson L; Endocrine Society. Pituitary incidentaloma: an endocrine society clinical practice guideline. J Clin Endocrinol Metab. 2011;96:894–904. 10.1210/jc.2010-1048 PMC5393422 21474686

[21] Hordejuk D Cheung YM Wang W. Long-term changes in the size of pituitary microadenomas. Ann Intern Med. 2023;176:298–302. 10.7326/M22-1728 36848656

