# Supplementary material for: Vestibular Schwannoma Management: Patient Perspectives and Health Literacy
Source: Otol Neurotol Open. 2024 Apr 26;4(2):e054. doi: 10.1097/ONO.0000000000000054 (PMC13585311; doi:10.1097/ONO.0000000000000054)
Supplement: Supplementary file 1 [file ono-4-e054-s001.pdf]

Supplemental Table S1: Patient-reported symptoms, hearing and tumor size at last follow-up

	**Treated**	**Observation**	**p-value**
Hearing			
Mean PTA (SD) in decibels	76.1 (40.6)<br>*(n = 74)*	42.6 (24.2)<br>*(n = 35)*	p < 0.001<br>95% CI: [-45.8, -21.1]
Mean WRS (SD), % correct*	57.7 (43.4)<br>*(n = 48)*	70.8 (30.0)<br>*(n = 32)*	p = 0.116<br>95% CI: [-3.3, 29.4]
Tumor Size at last MRI			
Mean largest dimension (SD) in millimeters**	10.0 (5.5)<br>*(n = 37)*	8.7 (5.7)<br>*(n = 45)*	0.295<br>95% CI: [-1.2, 3.8]
Interval Symptoms since Last Visit			
Decreased hearing, n (%)	75 (44.4%)<br>*(n = 159)*	28 (58.3%)<br>*(n = 48)*	0.219
Decline in balance, n (%)	63 (39.4%)<br>*(n = 160)*	12 (25.0%)<br>*(n = 48)*	0.099
Facial weakness, n (%)	20 (12.6%)<br>*(n = 159)*	4 (8.3%)<br>*(n = 48)*	0.607

Abbreviations: CI, confidence interval; MRI, magnetic resonance imaging; PTA, Pure Tone Audiogram; SD, standard deviation; WRS, Word Recognition Score

*Patients with untestable WRS at their last follow were excluded.

**Patients that underwent gross tumor resection with no measurable tumor remnants at last visit were excluded from analysis.
