# Supplementary material for: Vestibular Schwannoma Management: Patient Perspectives and Health Literacy
Source: Otol Neurotol Open. 2024 Apr 26;4(2):e054. doi: 10.1097/ONO.0000000000000054 (PMC13585311; doi:10.1097/ONO.0000000000000054)
Supplement: Supplementary file 2 [file ono-4-e054-s002.pdf]

Supplemental Table S2: Scores for each question on the Brief Health Literacy Screening (BHLS) tool, treated vs observation patients

	Treated (n = 160)	Observation (n = 48)	p-value
Average score, question 1 (SD)	4.58 (0.79)	4.42 (0.94)	0.294<br>95% CI: [-0.1, 0.5]
Average score, question 2 (SD)	4.58 (0.91)	4.46 (1.07)	0.497<br>95% CI: [-0.2, 0.5]
Average score, question 3 (SD)	4.68 (0.69)	4.50 (0.92)	0.212<br>95% CI: [-0.1, 0.5]

Abbreviations: BHLS, Brief Health Literacy Screen; CI, confidence interval; SD, standard deviation

Two patients in the observation group failed to complete question 2
