# Supplementary material for: Vestibular Schwannoma Management: Patient Perspectives and Health Literacy
Source: Otol Neurotol Open. 2024 Apr 26;4(2):e054. doi: 10.1097/ONO.0000000000000054 (PMC13585311; doi:10.1097/ONO.0000000000000054)
Supplement: Supplementary file 3 [file ono-4-e054-s003.pdf]

Appendix A: History, subjective symptom report, and Brief Health Literacy Screen (BHLS)

1) Have you undergone treatment for your vestibular schwannoma?
2) If so, where did the treatment occur, and when?
3) If not, having you experienced any of the following since you were last evaluated?
   a. Decline in hearing?
   b. Decline in balance or dizziness?
   c. New facial weakness, numbness or spasms?
4) When was the last time you had a MRI to evaluate your acoustic schwannoma?
5) When was the last time you saw a provider to discuss your history of a vestibular schwannoma?
   a. If it has been more than 2 years, what are the reasons for not returning?
   b. If it has been more than 2 years, Are you interested in re-establishing care for your vestibular schwannoma either at UTSW or at a different center?

Brief Health Literacy Screen:

6) How confident are you filling out medical forms by yourself?

   1 - Not at all
   2 - A little bit
   3 - Somewhat
   4 - Quite a bit
   5 - Extremely

7) How often do you have someone help you read hospital materials?

   1 - All of the time
   2 - Most of the time
   3 - Some of the time
   4 - A little of the time
   5 - None of the time

8) How often do you have problems learning about your medical condition because of difficulty understanding written information?

   1 - All of the time
   2 - Most of the time
   3 - Some of the time
   4 - A little of the time
   5 - None of the time
